# The CFTR M470V, Intron 8 Poly-T, and 8 TG-Repeats Detection in Chinese Males with Congenital Bilateral Absence of the Vas Deferens

**DOI:** 10.1155/2014/689185

**Published:** 2014-01-08

**Authors:** Qiang Du, Zheng Li, Yongfeng Pan, Xiaoliang Liu, Bochen Pan, Bin Wu

**Affiliations:** ^1^Department of Reproduction, ShengJing Hospital of China Medical University, Shenyang 110004, China; ^2^Department of Urology, RenJi Hospital, Shanghai Jiao Tong University School of Medicine, Shanghai 200127, China; ^3^Shenyang Institute of Criminal Technology, Shenyang 110004, China; ^4^Department of Clinical Genetics, ShengJing Hospital of China Medical University, Shenyang 110004, China; ^5^Department of Urology, ShengJing Hospital of China Medical University, Shenyang 110004, China

## Abstract

*Purpose*. To evaluate the significance of molecular detection of cystic fibrosis transmembrane conductance regulator (CFTR) M470V, intron 8 poly-T, and intron 8 TG-repeats in congenital bilateral absence of the vas deferens (CBAVD). *Methods*. Eighty-nine male patients with CBAVD and 103 healthy males were included in this study. Polymerase chain reaction was performed to amplify the polymorphic regions using primers from conserved regions. M470V was genotyped using real-time PCR by cycling probe. The exon 9 DNA sequence was determined using an automated sequencer. TG-repeats and poly-T were identified by direct sequencing analysis. *Results*. The 5T allele distribution was 0.32, 0.66 for 7T, and 0.02 for 9T in CBAVD males, respectively. In contrast, the 5T allele distribution was 0.03, 0.96 for 7T, and 0.01 for 9T in healthy control. Study of the polymorphisms of the upstream of exon 9 revealed a higher frequency of 5T allele in the CBAVD males. All cases with TG13T5 haplotype and TG12T5 homozygous led to CBAVD. The CFTR TG12T5-V470 variant haplotype was associated with CBAVD. *Conclusion*. The 5T allele of intron 8 of CFTR has clinically significant association with CBAVD. TG13T5 and TG12T5 homozygously led to CBAVD, and TG12T5-V470 may also lead to CBAVD.

## 1. Introduction

Congenital bilateral absence of the vas deferens (*CBAVD; OMIM 277180*) is found in 1% of infertile males and in up to 6% of those with obstructive azoospermia [1]. Extensive studies in western populations suggest that 75% of CBAVD cases are associated with mutations in the cystic fibrosis transmembrane conductance regulator (*CFTR; OMIM 602421*) gene, particularly the F508del, 5T, and R117H mutations [2]. However, prevalence of CFTR mutations among normal and infertile Chinese males is not well investigated. CBAVD may also be related to poly-T, TG-repeats, or M470V polymorphisms in the CFTR gene [3, 4]. Shorter poly-T tracts in CFTR nontranslated regions are associated with reduced levels of normal CFTR mRNA and protein [5]. Individuals harboring the T5 allele exhibit aberrant mRNA splicing of CFTR exon 9, generating a subset of transcripts that lack this exon and specify a nonfunctional CFTR protein [6, 7]. The incidence of the 5T allele in the healthy Chinese population is known to be 0.0380 [8], but the distribution of the poly-T, TG-repeat, and M470V polymorphisms among Chinese CBAVD patients has not been described. Moreover, it remains unclear whether M470V and (TG)mTn are risk factors for CBAVD in Chinese males. In this study, the distribution of (TG)mTn among normal Chinese males and Chinese CBAVD patients was assessed using DNA sequencing to explore the relationships between CBAVD and the TG-repeat, poly-T, and M470V polymorphisms in the CFTR gene.

## 2. Materials and Methods

### 2.1. Sample Collection

This study was approved by the Medical Ethics Committee of Shengjing Hospital of China Medical University (Shenyang, China). All patients gave written informed consent. Eighty-nine male patients with unambiguous clinical diagnoses of CBAVD and 103 normal male participants who underwent assisted reproductive treatment owing to female infertility were enrolled in the study. They were treated at the Center for Assisted Reproduction of Shengjing Hospital (Shenyang, China) from Aug. 2008 to Jan. 2012. Screening criteria for CBAVD patients were as follows: (1) no signs or symptoms of cystic fibrosis (CF) other than primary infertility; (2) normal bilateral testicular size with bilateral absence of the vas deferens; (3) absence of detectable sperm as determined by 3 routine semen exams using centrifugal sedimentation microscopy; absence of sperm in the urinary sediment following ejaculation (thus retrograde ejaculation has been excluded), acidic semen pH, and significantly lower quantitative of seminal fructose and glycosidase than normal; (4) normal levels of reproductive hormones and karyogram; and (5) recovery of sperm by aspirating from the epididymis or testis. Screening criteria for normal fertile male participants were as follows: (1) underwent assisted reproductive treatment due to female infertility; (2) normally developed bilateral testis; (3) normal routine semen test results; (4) normal karyogram.

### 2.2. Genomic DNA Extraction

Peripheral blood samples (2 mL) were collected from each subject and were anticoagulated with ethylenediaminetetraaceticacid (EDTA). Genomic DNA was extracted using the automated BioRobot EZ1 Workstation (Qiagen, Hilden, Germany) with the EZ1 DNA blood 200 *μ*L kit (Qiagen, Hilden, Germany). DNA concentrations were measured using UV spectrophotometry. DNA purity was validated by measuring the A_260_/A_280_ ratio, and samples measuring between 1.8 and 2.0 were retained. Samples failing this test were reextracted. DNA samples then were identified with serial numbers and stored at −80°C until subsequent analyses.

### 2.3. Detection of M470V in the CFTR Gene with Real-Time PCR

The following primers flanking the M470V region of the CFTR gene were designed with *Primer 5.0 software:* sense 5′-CTTCTGCTTAGGATGATAATTGG-3′ and antisense 5′-GCTTTGATGACGCTTCTGTA-3′. Primers and corresponding fluorescent probes were synthesized by TaKaRa Biotechnology (Dalian, China). The probes were 5′-(FAM) cttctaatg (A) tg (Eclipse)-3′and 5′-(HEX) tctaatg (G) tga (Eclipse)-3′. The cycling probe method [9] was used to genotype samples for the M470V polymorphism. PCR products were analyzed by 3% agarose gel electrophoresis to confirm that specific bands had been amplified by the synthesized primers and probes. Real-time PCR products were sequence-verified to ensure that the primers and probes were effective in the detection of M470V. PCR was performed using a reaction solution specified by the CycleavePCR core kit instructions (TaKaRa Biotechnology). Components and conditions of the real-time PCR are briefly described and were the following: 1 *μ*L of the DNA sample, 2.5 *μ*L 10× Cycleave PCR Buffer, 3 *μ*L dNTP mixture (2.5 mM each), 5 *μ*L Mg solution (25 mM), 0.5 *μ*L each primer pair (10 uM), 1 *μ*L each probe pair (3uM), 0.5 *μ*L Tli Rnase H II (200 U/*μ*L), 0.25 *μ*L TaKaRa Ex Taq HS (5 U/*μ*L), and 9.75 *μ*L RNase free dH2O. After initial denaturation at 95°C for 30 sec, the PCR system was given 50 cycles of denaturation at 95°C for 5 sec, annealing at 57°C for 15 sec, and extension at 72°C for 34 sec and 5 min. Samples were analyzed using an ABI 7500 Sequence Detection System (Applied Biosystems) in combination with a postreaction melting curve analysis.

### 2.4. Sequence Detection of Poly-T and TG-Repeats in the CFTR Gene

The following primers flanking the poly-T and TG-repeat regions of the CFTR gene were designed with Primer 5.0 software: sense 5′-TGGGCAAATATCTTAGTTT-3′ and antisense 5′-GAGTACCAAGAAGTGAGAA-3′. Primers were synthesized and sequenced by TaKaRa Biotechnology. The reaction components and conditions were the following: 1 *μ*L of the DNA sample, 25 *μ*L 10× Ex Buffer, 0.5 *μ*L each primer pair (3 uM), 2.5 *μ*L dNTP (2.5 Mm each), and 20.5 *μ*L dH2O. The initial denaturation at 94°C for 60 sec was followed by 40 cycles of denaturation at 94°C for 30 sec, annealing at 55°C for 30 sec, and extension at 72°C for 30 sec and 120 sec. PCR products (5 *μ*L) migrated through an agarose gel as a single band of approximately 318 bp. Amplicons were purified using shrimp alkaline phosphatase and exonuclease I (TaKaRa Biotechnology) and subsequently were sequenced. Sequencing results were analyzed using Chromas v.1.62 software, and samples were individually confirmed manually. Paired-end sequencing was performed to verify sequences that could not be confirmed by the above approach.

### 2.5. Statistics

Data were evaluated with Pearson's chi-squared test and Fisher's exact test using SPSS v.13.0 software. Significance was assessed at *P* < 0.05.

## 3. Results

The clinical characteristics and genotypes of the patients in this study are shown in Table 1. The MV genotype was the most prevalent, with a frequency of 44.66% among normal Chinese males, followed by the VV genotype, with a frequency of 35.92%. The MM genotype was the least common, with a frequency of 19.42%. The distribution of MM, MV, and VV genotypes among normal males was consistent with the Hardy-Weinberg equilibrium (*P* = 0.4065). The V470 allele occurred most often among normal male participants, with a frequency of 58.25%. The distribution ratio of M470 and V470 alleles among normal males was approximately 0.7 : 1. We detected no statistically significant differences in the M470V genotype distributions or allele frequencies between Chinese CBAVD patients and fertile male participants (*P* = 0.3177 and *P* = 0.1615, resp.) (Table 2).

With regard to poly-T polymorphisms, T7 was the most common allele among normal Chinese male participants, with a frequency of 96.12% (Table 3). Of the TG-repeats, TG12 was most frequently represented among normal Chinese males (frequency 55.83%), followed by TG11 (43.20%). The allele frequency distributions of the T5 and TG13 alleles significantly differed between the two groups (*P* < 0.001; see Table 3). T7/T7 was the most commonly detected haploid genotype among normal Chinese males (92.23%); all T5/T5 were CBAVD (Table 4). TG12T7 was the most commonly detected haploid genotype among normal Chinese males (52.43%), followed by TG11T7 (42.72%) (Table 5). The frequency distributions of the TG13T5 and TG12T5 haploid genotypes differed significantly (*P* < 0.001). Among 89 CBAVD patients, 14 individuals were heterozygous for the TG13T5 haploid genotype and 1 individual was a TG13T5 homozygote. None of the 103 normal male participants harbored the TG13T5 haplotype. All 5 TG12T5 homozygotes identified were CBAVD patients.

In addition, the frequency distributions of the TG12T5-V470 haploid genotypes differed significantly (*P* < 0.001) (only the homozygote of (TG)mTn and Tn could be analyzed in the genotype frequencies of haploid) (Table 6). Our results suggest that individuals with a TG12T5-V470 haploid genotype have a significantly higher risk of CBAVD (*P* < 0.001). In the entire study population, none of the individuals carried the TG13T5-V470 haploid genotype particularly.

## 4. Discussion

The CFTR gene was originally cloned and identified by Riordan et al. in 1989 [10]. CFTR is located on chromosome 7q31.2 and is 188,704 bp in length [11], spanning 26 introns and 27 exons. The CFTR gene encodes a 6,132-bp mRNA that specifies the 1,480-residue CFTR protein. The CFTR protein has a relative molecular weight of approximately 168,173. The in vivo function of the CFTR protein is determined by its genotype [12].

CBAVD, a CF-related disorder (*OMIM 219700*) [13], is an important congenital cause of male infertility and obstructive azoospermia, which correlated closely with specific CFTR mutations in western populations, mainly R117H, delF508, and 5T, which are distributed differently in different subgroups of people [2]. CFTR gene analyses have focused primarily on the intron 8 polymorphism, poly-T (IVS8 poly-T), as well as TG-repeats, and the M470V allele. However, only 4 studies have been performed to investigate the poly-T polymorphism and congenital bilateral absence of the vas deferens in Chinese patients [14–17].

Interestingly, although the poly-T polymorphisms in those studies were similar, the frequency data differed greatly from each other and this could be because those studies were performed with subjects from different regions of China. The study [16] reported that the frequency of 5T was about 44.5% in Chinese patients with congenital bilateral absence of the vas deferens and 13.46 in normal subjects, which were substantially higher than the findings reported [15]. Radpour et al. [3] found a 5T allele frequency of 25.94% among 106 CBAVD patients. In contrast, the authors did not detect the 5T allele in a population of 43 normal male participants. The findings suggested that the distribution of poly-T polymorphism could vary in different regions, and more studies should be performed to further investigate this point. In our study, normal healthy Chinese people carry 7T or 9T most frequently. Shorter Tn tracts are associated with reduced levels of normal CFTR mRNA. CFTR mRNA splicing at exon 9 can be affected in 5T individuals, resulting in an mRNA subpopulation missing exon 9 that specifies a nonfunctional CFTR protein. The missing exon 9 in 5T individuals often translates to CBAVD without other CF symptoms.

The 5T allele polymorphism varies considerably by geographic location, ranging from 0.0013% to 43.7% [18–22]; this polymorphism is particularly variable among non-Caucasian populations. We detected a T5 allele frequency of 32.02% among Chinese CBAVD patients, which is higher than that in the other report [3]. We also detected the T5 allele among normal Chinese males at a frequency of 3.40%. The data is the most approximation to the previous report  [8] suggesting that the T5 allele is insufficient to cause CBAVD. However, all the subjects carrying homozygous 5T were patients with congenital bilateral absence of the vas deferens in the present study, while no subject with normal fertility was found to carry the homozygous 5T genotype, which further confirmed that shorter poly-T tracts are associated with more severe exon 9 splicing defects and a consequent functional decline in the CFTR protein. It is possible that the function of the CFTR protein is uniformly reduced among Chinese individuals compared with Caucasians.

Our results suggest that poly-T polymorphisms further affect the splicing of exon 9 when they precede TG-repeats. Compared with TG10 in a T7 background, Cuppens reported that the TG11 and TG12 alleles generated CFTR transcripts missing exon 9 at higher rates of 2.8- and 6-fold, respectively [23]. TG-repeats determine CFTR protein function and work in synergy with poly-T [24]. Longer TG-repeat tracts are associated with shorter poly-T tracts and elevated probabilities of exon 9 skipping [6]. Exon 9 encodes a portion of the CFTR protein's first nucleotide binding domain [25], and transcripts lacking exon 9 specify misfolded and dysfunctional CFTR chloride channels [26]. Fluid secretion is necessary for the normal development of renal tubules. If fluid secretion is blocked during embryonic development, renal tubular dysgenesis and early degradation occur [11, 27]. Because the vas deferens is highly sensitive to abnormal CFTR protein function, an increased risk of CBAVD maybe results from exon 9-null transcripts [28].

Fourteen of the CBAVD patients in our study were TG13T5 heterozygotes, supporting the theory that TG-repeats work with poly-T synergistically to precipitate CBAVD. We identified one TG13T5 homozygous CBAVD patient descended from a consanguineous union. To assess whether this genotype resulted from a genetic mutation, we recommended genetic testing of the patient's parents. In subsequent studies, we intend to expand our sample size to investigate the incidence of TG13T5. Our results confirm the importance of a follow-up study for the offspring of CBAVD patients who were conceived by intracytoplasmic sperm injection (ICSI) assisted reproductive treatment, to determine whether offspring carrying TG13T5 manifest as CBAVD. Five CBAVD patients in our study were TG12T5 homozygotes, whereas this genotype was not detected among the normal male participants. This finding confirms that TG-repeats function synergistically with poly-T to produce CBAVD.

Although no association between M470V and congenital bilateral absence of the vas deferens has been reported, M470V also may interact synergistically with poly-T and TG-repeats [29]. Our results show that the M470V allele alone is insufficient to produce CBAVD, but this polymorphism may contribute to CBAVD risk in individuals with short poly-T and long TG-repeat tracts, which is in agreement with the findings reported by Tomaiuolo et al. [30]. TG12T5-V470 haploid individuals are at greater risk of CBAVD. In our entire study population, no individuals carried the TG13T5-V470 haploid genotype; it is possible that this haploid genotype may cause a more severe phenotype. Therefore, examinations which could identify the genotypes of poly-T, TG repeats, and M470V of the CFTR gene in Chinese patients with CBAVD should and could be performed to avoid the inheritance of this disease to their children, thanks to the development of in vitro fertilization and ICSI technology [31]. Additional studies of larger populations are needed to address this question.

## Figures and Tables

**Table 1:** CFTR genotypes in Chinese CBAVD patients and males with normal fertility.

M470V genotypes	Poly-T genotypes	(TG_*m*_) T_*n*_ genotypes	CBAVD	Normal fertility males
M/M	T5/T5	TG13T5/TG13T5	1	—
M/M	T5/T7	TG13T5/TG12T7	6	—
M/M	T5/T9	TG13T5/TG11T9	1	—
M/M	T5/T9	TG12T5/TG11T9	1	—
M/M	T7/T7	TG11T7/TG12T7	1	—
M/M	T7/T7	TG11T7/TG11T7	1	—
M/M	T7/T7	TG12T7/TG12T7	10	19
M/M	T7/T7	TG12T7/TG13T7	—	1
M/V	T5/T5	TG13T5/TG12T5	7	—
M/V	T5/T5	TG12T5/TG12T5	1	—
M/V	T5/T7	TG12T5/TG12T7	13	4
M/V	T5/T7	TG12T5/TG11T7	2	—
M/V	T5/T9	TG12T5/TG10T9	1	—
M/V	T7/T7	TG11T7/TG12T7	15	32
M/V	T7/T7	TG11T7/TG13T7	—	1
M/V	T7/T7	TG12T7/TG12T7	6	9
V/V	T5/T5	TG12T5/TG12T5	4	—
V/V	T5/T7	TG12T5/TG12T7	1	2
V/V	T5/T7	TG12T5/TG11T7	6	1
V/V	T7/T7	TG11T7/TG12T7	6	10
V/V	T7/T7	TG11T7/TG11T7	5	22
V/V	T7/T7	TG12T7/TG12T7	—	1
V/V	T7/T9	TG11T7/TG11T9	1	—
V/V	T7/T9	TG11T9/TG12T7	—	1

**Table 2:** Genotypes and allele frequencies at M470V polymorphic site.

	CBAVD (frequencies) (2*n* = 178)	Control (frequencies) (2*n* = 206)	*P*
Frequencies of individuals with genotypes			
MM	21/89 (23.60%)	20/103 (19.42%)	0.3177
VV	23/89 (25.84%)	37/103 (35.92%)	
MV	45/89 (50.56%)	46/103 (44.66%)	
Allele frequencies at M470V polymorphic site			
M	87/178 (48.88%)	86/206 (41.75%)	0.1615
V	91/178 (51.12%)	120/206 (58.25%)	

**Table 3:** Allele frequency of poly-T and TG-repeats polymorphisms.

	CBAVD (frequencies) (2*n* = 178)	Control (frequencies) (2*n* = 206)	*P*
Allele frequency of poly-T			
T5	57/178 (32.02%)	7/206 (3.40%)	<0.001
T7	117/178 (65.73%)	198/206 (96.12%)	<0.001
T9	4/178 (2.25%)	1/206 (0.49%)	0.286
Allele frequency of TG-repeats			
TG10	1/178 (0.56%)	0/206	0.464*
TG11	46/178 (25.84%)	89/206 (43.20%)	<0.001
TG12	115/178 (64.61%)	115/206 (55.83%)	0.080
TG13	16/178 (8.99%)	2/206 (0.97%)	<0.001

*Fisher's exact test.

**Table 4:** Frequencies of genotypes at poly-T polymorphic site.

Type/number (*n*)	Frequencies of genotypes at poly-T polymorphic site				
	T5/T5	T5/T7	T7/T7	T5/T9	T7/T9
CBAVD (*n* = 89)	13/89 (14.61%)	28/89 (31.46%)	44/89 (49.44%)	3/89 (3.37%)	1/89 (1.12%)
Control (*n* = 103)	0/103	7/103 (6.80%)	95/103 (92.23%)	0/103	1/103 (0.97%)
*P*	<0.001	<0.001	<0.001	0.195	1.000*

*Fisher's exact test.

**Table 5:** Linkage of poly-T and TG-repeats.

Linkage haplotypes	CBAVD (frequencies) (2*n* = 178)	Control (frequencies) (2*n* = 206)	*P*
TG10T9	1/178 (0.56%)	0/206	0.464*
TG11T7	43/178 (24.16%)	88/206 (42.72%)	<0.001
TG11T9	3/178 (1.69%)	1/206 (0.49%)	0.515
TG12T7	74/178 (41.57%)	108/206 (52.43%)	0.034
TG12T5	41/178 (23.03%)	7/206 (3.40%)	<0.001
TG13T7	0/178	2/206 (0.97%)	0.501*
TG13T5	16/178 (8.99%)	0/206	<0.001

*Fisher's exact test.

**Table 6:** Linkage of poly-T, TG-repeat, and M470V.

Linkage haplotypes	CBAVD (frequencies) (2*n* = 88)	Control (frequencies) (2*n* = 114)	*P*
TG11T7-M470	3/88 (3.41%)	0/114	0.162
TG11T7-V470	23/88 (26.14%)	55/114 (48.25%)	<0.001
TG11T9-M470	2/88 (2.27%)	0/114	0.189*
TG11T9-V470	1/88 (1.14%)	1/114 (0.88%)	1.000*
TG12T7-M470	27/88 (30.68%)	39/114 (34.21%)	0.596
TG12T7-V470	7/88 (7.95%)	15/114 (13.16%)	0.239
TG12T5-M470	1/88 (1.14%)	0/114	0.436*
TG12T5-V470	15/88 (17.05%)	3/114 (2.63%)	<0.001
TG13T7-M470	0/88	1/114 (0.88%)	1.000*
TG13T5-M470	9/88 (10.23%)	0/114	0.002
TG13T5-V470	0/88	0/114	—

*Fisher's exact test.
